# Flowering phenology and potential pollen emission of three *Artemisia* species in relation to airborne pollen data in Poznań (Western Poland)

**DOI:** 10.1007/s10453-015-9397-z

**Published:** 2015-07-17

**Authors:** Paweł Bogawski, Łukasz Grewling, Agata Frątczak

**Affiliations:** Laboratory of Aeropalynology, Faculty of Biology, Adam Mickiewicz University, Umultowska 89, 61-614 Poznan, Poland; Department of Climatology, Faculty of Geographical and Geological Sciences, Adam Mickiewicz University, Dzięgielowa 27, 61-680 Poznan, Poland; Department of Plant Taxonomy, Faculty of Biology, Adam Mickiewicz University, Umultowska 89, 61-614 Poznan, Poland

**Keywords:** Pollen production, Aeroallergen, *Artemisia vulgaris*, *Artemisia absinthium*, *Artemisia campestris*

## Abstract

*Artemisia* pollen is an important allergen in Europe. In Poznań (Western Poland), three *Artemisia* species, *A. vulgaris*, *A. campestris* and *A. absinthium*, are widely distributed. However, the contributions of these species to the total airborne pollen are unknown. The aim of the study was to determine the flowering phenology and pollen production of the three abovementioned species and to construct a model of potential *Artemisia* pollen emission in the study area. Phenological observations were conducted in 2012 at six sites in Poznań using a BBCH phenological scale. Pollen production was estimated by counting the pollen grains per flower and recalculating the totals per inflorescence, plant and population in the study area. Airborne pollen concentrations were obtained using a Hirst-type volumetric trap located in the study area. *Artemisia vulgaris* began to flower the earliest, followed by *A. absinthium* and then *A. campestris*. The flowering of *A. vulgaris* corresponded to the first peak in the airborne pollen level, and the flowering of *A. campestris* coincided with the second pollen peak. The highest amounts of pollen per single plant were produced by *A. vulgaris* and *A. absinthium*. *A. campestris* produced considerably less pollen, however, due to its common occurrence, it contributed markedly (30 %) to the summation of total of recorded pollen. *A. vulgaris* is the most important pollen source in Poznań, but the roles of two other *Artemisia* species cannot be ignored. In particular, *A. campestris* should be considered as an important pollen contributor and likely might be one of the main causes of allergic reactions during late summer.

## Introduction

*Artemisia* pollen is one of the most important aeroallergens, particularly in Central Europe, where the sensitization rate ranges from 10.6 % in Austria to 44.3 % in Hungary (Burbach et al. 2009). In Poland, this taxon is considered the third most important source (after birch and grasses) of allergenic pollen, and the prevalence of pollinosis to *Artemisia* allergens is approximately 10–15 % (Stach et al. 2007; Burbach et al. 2009). The highest concentrations of *Artemisia* pollen are usually observed in Poznań (Western Poland) between the middle of July and the beginning of September (Stach et al. 2007); however, a significant expansion of *Artemisia* pollen seasons has been observed in recent years (Bogawski et al. 2014).

Pollen grains produced by different *Artemisia* species are very similar, i.e. trizonocolporate and small (18–24 µm), with scabrate exine thickening between neighbouring furrows (Accorsi et al. 1991). Under a light microscope, they are practically indistinguishable; in standard aerobiological monitoring, all collected *Artemisia* pollen grains are therefore considered together as ‘*Artemisia* pollen type’. However, worldwide there are over 50 species of *Artemisia* with different ecological and physiological requirements, different times of flowering and pollen release and differences in both the size and structure of inflorescences (Tutin 1972; Laursen et al. 2007; Grewling et al. 2012).

Presumably, pollen production, an important issue from both biological and allergological points of view, could also vary by *Artemisia* species. In this context, it is worth noting that three *Artemisia* species, *A. vulgaris*, *A. campestris* and *A. absinthium*, are widely distributed in Poznań (Jackowiak 1993) and may affect sensitized patients in the city. However, there are no comprehensive data about their flowering phenology or pollen production; consequently, there is no estimate of the contributions of different *Artemisia* species to the airborne pollen curve.

To fill this gap in knowledge, we performed an experimental field study in Poznań, in the 2012 *Artemisia* growing season. The study was focused on examining the flowering cycle of three *Artemisia* species (*A. vulgaris*, *A. campestris* and *A. absinthium*). The final goal of the study was to construct, based on phenological and pollen production data, a model of the potential *Artemisia* pollen emission in the Poznań area.

## Materials and methods

### Study area and climate

Poznań is a medium-sized European city located in Western Poland, Central Europe (16°E, 52°W). The mean annual temperature in the region is 8.2 °C, and the mean annual precipitation reaches approximately 500 mm (Woś 2010). The study was conducted in the northern part of Poznań, approximately 8 km from the city centre. This area is dominated by new housing estates, the university campus, agricultural fields and small patches of forests. In addition, barren lands, meadows and abandoned sites, which offer suitable habitats for *Artemisia* species (Gucker 2007; Barney and DiTommaso 2003), are common in the investigated area.

### Aerobiological and meteorological data

**Fig. 1:**
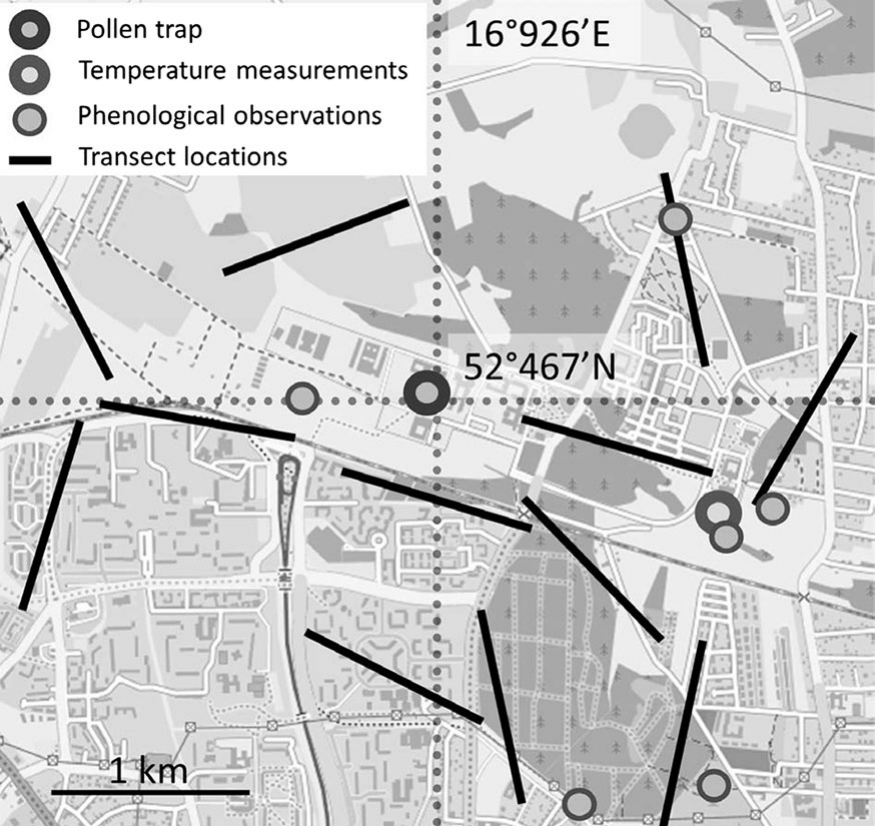
Study area and the transect locations. Forests (*irregular dark grey*), buildings (*regular dark grey*), agricultural fields and wastelands (*grey and light grey*). Basemap: © OpenStreetMap (and) contributors, CC-BY-SA

Daily average *Artemisia* pollen data were collected using a 7-day volumetric spore trap of the Hirst design (Hirst 1952) situated 18 m above ground level, on the roof of the Collegium Biologicum, Adam Mickiewicz University in Poznań (Fig. 1). Pollen grains were identified at 400× magnification under a light microscope. Counting of the pollen grains was performed according to the recommendations of the Spanish Aerobiology Network (Galán et al. 2007). Daily average *Artemisia* pollen counts were converted to grains per cubic metre of air (pollen/m^3^).

Mean daily temperature data were recorded by a HOBO U23-001 data logger sheltered in a standard Stevenson screen located 1 km from the pollen trap. Daily precipitation totals recorded at the Poznań-Ławica airport station (approximately 8 km from the study area) were obtained by the NOAA (National Oceanic and Atmospheric Administration) server.

### Phenological data

The most common *Artemisia* species in Poland are *A. vulgaris*, *A. campestris* and *A. absinthium* (Zając and Zając 2001). Some other species are quite frequent regionally, i.e. *A. annua*, *A. scoparia* and *A. austriaca* that occur mainly in south-eastern Poland, around 500 km from Poznań (Fijałkowski 1967; Zając and Zając 2001; Mirek et al 2002). The remaining species are rare. Among them, there are several ornamental species (*A. dracunculus*, *A. ludoviciana*, *A. rupestris*, *A. lanata*, *A. schmidtiana*, *A. frigida*, *A. stelleriana*) that are not spread spontaneously but only planted (excluding *A. dracunculus*). Some of *Artemisia* species are ephemeral (*A. biennis*, *A. maritima*, *A. verlotiorum* and *A. tournefortiana*) in Poland (found only sporadically) (Mirek et al. 2002). In addition, two autochthonous *Artemisia* spp. are considered rare and critically endangered (*A. eriantha* and *A. pontica*, respectively) (Zarzycki and Mirek 2006).

In Poznań, seven species of *Artemisia* have been recorded: *Artemisia vulgaris*, *A. absinthium*, *A. campestris*, *A abrotanum*, *A austriaca*, *A. annua* and *A. scoparia*. Two of them occurred sparsely, i.e. *A. abrotanum* was observed at one site and *A. austriaca* at two sites in Poznań. The next two species, *A. scoparia* and *A. annua*, occurred in Poznań till the year 1980, but they have not been observed later (no contemporary records) (Jackowiak 1993). In contrast, *A. vulgaris* and *A. campestris* were frequently recorded, almost in every 1 km^2^ of the city area, and *A. absinthium* was slightly less frequent, especially in the city centre (Jackowiak 1993). The average distance from which the pollen can be transported to particular point (here: pollen trap) varies according to different authors from 800 m (Bricchi et al. 2000) to 30–100 km (Faegri and Iversen 1989; Avolio et al. 2008) from the source. Although anemophilous, *Artemisia*, pollen has a low ability for wind transport, for instance, its airborne concentration rapidly decreases along the height above ground level (Rantio-Lehtimaki et al. 1991; Spieksma et al. 2000). Taking into consideration the population size and distance from the study area, only *A. vulgaris*, *A. campestris* and *A. absinthium* may constitute an effective source of pollen in Poznań. Therefore, only these three species have been eventually chosen for phenological observations.

Phenological observations were made every 2–3 days from the beginning of July till the day when the last plant has finished flowering (the first week of September) at six locations within a radius of 2 km from the pollen monitoring station (Fig. 1). At every site, 20 mature individual plants of each *Artemisia* species were selected for further observation; however, due to the lack of a sufficient number of *A. absinthium*, the phenological phases for this species were examined at only three sites. In total, 300 individual plants of *Artemisia* spp. were observed, i.e. 120 individuals of *A. vulgaris*, 120 of *A. campestris* and 60 of *A. absinthium*.

The phenological phases were determined on the basis of the BBCH scale (Hess et al. 1997). The following growth stages were observed: beginning of flowering (BBCH 61: 10 % of flowers open), full flowering (BBCH 65: 50 % of flowers open), flowering finishing (BBCH 67: majority of inflorescences fallen or dry) and end of flowering (BBCH 69). The duration of the whole flowering season and of the full flowering phase were also calculated. Phenological records (sampling dates) were expressed as the day of the year (DOY) counted from 1 January. The relative frequencies (%) of selected phenophases of each *Artemisia* species on certain days were calculated.

### Pollen production data

To estimate the number of pollen grains per flower, the method proposed by Cruden (1977) and developed by Hidalgo et al. (1999) was applied. Inflorescences were collected at the six previously described phenological sites. At every site, 15 mature inflorescences were collected from the top part of the central shoot of each *Artemisia* species (similar to the phenological observations, inflorescences of *A. absinthium* were investigated at three sites). In total, 225 inflorescences were collected (90 of each of *A. vulgaris* and *A. campestris* and 45 of *A. absinthium*). From every inflorescence, a single isolated flower (before anthesis) was crushed and mixed in 100 µl of distilled water. Next, 10 µl of concentrate was deposited on a microscopic slide, and the number of pollen grains was counted under a light microscope (magnification 200×). The examination was repeated three times per sample, and the obtained values were averaged. To calculate the potential pollen production of each *Artemisia* species, the number of counted pollen grains per flower was multiplied by the mean number of flowers per inflorescence and mean number of inflorescences per plant.

These final values were used to estimate the potential pollen production of each *Artemisia* species in the study area via multiplication by the number of *Artemisia* individuals in the study area. As the species density may be accurately calculated by investigating 1 % of the whole area (Barbour et al. 1987), we sampled a 120,000 m^2^ area by means of the simple random coordinate method (Elzinga et al. 1998). Random coordinates for the starting points of twelve transects (10 m × 1000 m) and compass bearings were generated by a random number generator in Rundom Pro 3.14 (Jadwiszczak 2009). In the selected transects, all *Artemisia* individuals were counted and multiplied by 100 to estimate the number of *Artemisia* individuals within the whole investigated area.

### Statistical analyses

To detect statistically significant differences in the flowering phases and pollen production between the three *Artemisia* species, the nonparametric Kruskal–Wallis ANOVA with Newman and Keuls *posthoc* test was applied. Spearman’s correlation coefficient was used to analyse the relationships between aerobiological and phenological data. Spearman coefficient was calculated between the phenological record [relative frequencies (%) of individuals of each *Artemisia* species in the BBCH 61 and BBCH 65 phases] collected at certain sampling day and the 3-day mean of daily *Artemisia* pollen concentration recorded before, during and after the day of phenological observation, in three analysed periods:

1. Whole flowering season (26 July–3 September),
2. First part of the flowering season (26 July–15 August),
3. Second part of the flowering season (16 August–3 September).

### Model of potential pollen emission

The pollen emission model has been inspired by the study performed by Hidalgo et al. (2003) and was determined by combining phenological observation data with potential pollen production data in the study area (see Chapter *Pollen production data*). Every model was presented graphically in the form of triangle. Each triangle corresponds to the theoretical pollen emission of each *Artemisia* spp.

The vertices of a triangle were determined by phenological data: minimum dates of the onset (BBCH 61) and maximum dates of the end (BBCH 69) of flowering correspond to the beginning and the end of the pollen emission period (the lower left and right vertices of a triangle, respectively). Subtracting the date of the beginning from the date of the end of the pollen emission period resulted in the length of the base of a triangle (*b* in equation below). The top vertex of a triangle corresponds to the day with maximum number of individuals in full flowering (BBCH 65), as we assumed that the highest potential pollen emission occurred on the day with the highest frequency of *Artemisia* individuals in the BBCH 65 phase.

The area of triangle (*A*_t_) reflects the estimated value of potential pollen emission of each *Artemisia* species. The vertical height of a triangle was determined from the transformed formula for the area of a triangle as follows:

$$h = \frac{{2 \times A_{\text{t}} }}{b}$$

where *h* is vertical height of a triangle, *A*_t_ is the area of a triangle, i.e. the fraction of pollen (%) potentially produced (multiplication of the number of pollen produced by one plant by the estimated total number of plants in study area) by one particular species related to the general *Artemisia* pollen production in study area, and *b* is the length of the base of a triangle (length of pollen release period).

Then, the general pollen emission model curve was constructed by adding the values of the pollen emission curves of the individual species. The general model curve was normalized by attributing a value of 1 to the theoretical peak day. This model curve was compared with the observed airborne pollen concentrations in the 2012 pollen season and with the 8-year average (2005–2012). The 3-day moving average curves of pollen concentration were calculated and normalized in the same way as the model curve. The theoretical and observational data sets were compared with each other by calculating the coefficients of determination (*r*^2^).

## Results

### Aerobiological data

**Fig. 2:**
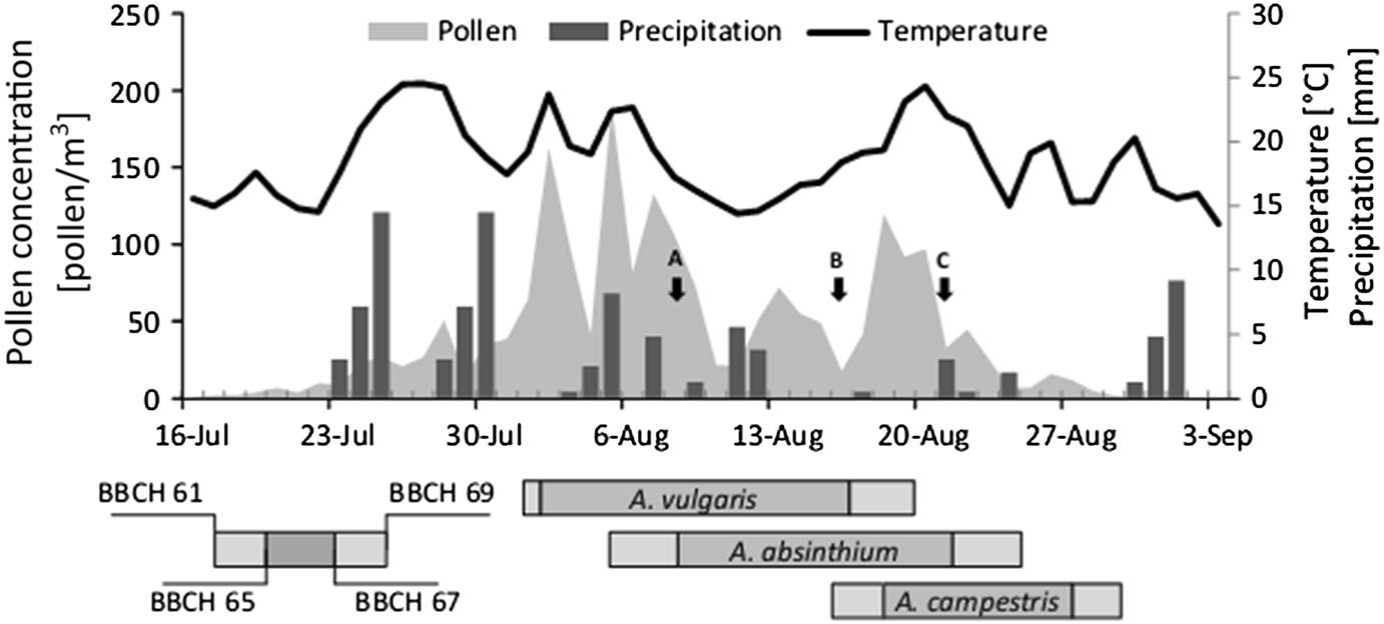
Temporal variation in flowering phenology of *Artemisia vulgaris*, *A. absinthium* and *A. campestris* (*horizontal bars*), airborne *Artemisia* pollen concentration, daily sum of rain and daily average air temperature patterns in 2012. Mean was the statistics used to present the onset dates of particular phenophases (see Table 1). The day with the highest number of individuals in full flowering is marked by *arrow* (**A** *A. vulgaris*, **B** *A. absinthium*, **C** *A. campestris*)

The first *Artemisia* pollen grains were recorded on 5 July, 2012 (DOY 187) (Fig. 2); however, during the next 2 weeks, the daily average pollen concentration never reached 10 pollen/m^3^. A strong increase in the *Artemisia* pollen level was observed at the end of July, which corresponded with an increase in the daily average air temperature. At the beginning of August, the daily average pollen concentrations exceeded 100 pollen/m^3^. During the next week, a very high *Artemisia* pollen level was observed in the air, with a maximum daily concentration recorded on 5 August (191 pollen/m^3^). In the second week of August, a slight decrease in the *Artemisia* pollen level was noted. The daily average pollen level increased again on 18 August (231 DOY) reaching 120 pollen/m^3^; however, the level was ranged between 26 to 97 pollen/m^3^ in the following few days. At the end of August, the *Artemisia* pollen level decreased below 10 pollen/m^3^. During September, the pollen level never exceeded >2 pollen/m^3^, and the last *Artemisia* pollen grains were observed on 29 September (273 DOY).

### Flowering phenology

*Artemisia vulgaris* began to flower first (mean = 215 DOY, 2 August), followed by *A. absinthium* (mean = 219 DOY, 6 August) and *A. campestris* (mean = 230 DOY, 17 August) (Table 1; Fig. 2). The duration of the full flowering phase (BBCH 65) varied from 7 to 15 days (for *A. campestris* and *A. vulgaris*, respectively). The days with the highest number of individuals in BBCH 65 occurred on 9 August (*A. vulgaris*), 17 August (*A. absinthium*) and 22 August (*A. campestris*) (Fig. 2). Similar to previous phases, phase BBCH 67 (finishing of flowering) began significantly earlier (*p* < 0.05) in *A. vulgaris* than in *A. absinthium* and *A. campestris* (4 and 10 days, respectively) (Table 1). The end of flowering (BBCH 69) of *A. vulgaris* was recorded on 20 August (233 DOY), and *A. campestris* reached this phase 4 days later. *A. campestris* finished flowering on 29 August (mean = 242 DOY). The duration of the whole flowering period (BBCH 61–BBCH 69) of *A. campestris* (12 days) was significantly shorter (*p* < 0.05) than those of the two other *Artemisia* species (18 days).

**Table 1:** Characteristics of *Artemisia* spp. flowering period obtained by phenological observations according to BBCH scale

Taxon	Descriptive statistic	BBCH 61 (DOY)	BBCH 65 (DOY)	BBCH 67 (DOY)	BBCH 69 (DOY)	Duration of BBCH 65 (days)	Duration of the whole flowering period (days)
*A. vulgaris*	Max	219	224	234	237	17	21
	Min	211	211	227	229	10	15
	Mean (*n* = 120)	215*	216*	230*	233*	15*	18
	SD	2.5	2.9	2.3	1.4	1.7	1.6
	CV (%)	1.1	1.4	1.0	0.5	9.4	11.0
*A. absinthium*	Max	232	232	240	243	16	23
	Min	211	211	227	232	5	11
	Mean (*n* = 60)	219*	222*	234*	237*	12*	18
	SD	4.9	5.7	3.9	2.6	2.5	2.8
	CV (%)	2.3	2.6	1.7	1.9	21.2	15.5
*A. campestris*	Max	237	240	243	246	10	16
	Min	221	224	234	237	3	9
	Mean (*n* = 120)	230*	232*	240*	242*	7*	12*
	SD	3.7	3.5	2.5	2.8	1.8	1.6
	CV (%)	1.6	1.5	1.1	1.2	24.7	13.6

The statistical differences were examined using Kruskal–Wallis ANOVA with Newman–Keuls *post* *hoc* test

* *p* value < 0.05

### Correlation between phenological and aerobiological data

The relative frequencies (%) of individuals of *A. vulgaris* and *A. absinthium* in phase BBCH 61 recorded during the whole flowering season were significantly correlated with the *Artemisia* airborne pollen concentration (*r* = 0.71 and *r* = 0.65, respectively, *p* < 0.01 for both) (Table 2). Similar results were obtained in relation to the BBCH 65 phase (*r* = 0.74, *p* < 0.01 and *r* = 0.57, *p* < 0.05, respectively). In contrast, the phenological data of *A. campestris* did not show a significant correlation with the *Artemisia* pollen concentration when examining the entire investigated season. However, in the second half of the flowering season (15 August–3 September), the relative frequencies of the *A. campestris* individuals in phases BBCH 61 and BBCH 65 revealed significant correlations with the aerobiological data series (*r* = 0.76 and *r* = 0.78, respectively, *p* < 0.05 for both).

**Table 2:** Spearman correlation coefficient between phenological and aerobiological data

Period	BBCH 61			BBCH 65		
	*A. vulgaris*	*A. absinthium*	*A. campestris*	*A. vulgaris*	*A. absinthium*	*A. campestris*
*Whole flowering season*						
26 July–3 September	0.710**	0.650**	0.354	0.741**	0.567*	0.044
*First part of the flowering season*						
26 July–15 August	0.687	0.371	0.307	0.429	0.405	0.192
*Second part of the flowering season*						
16 August–3 September	–	0.408	0.761*	0.686	0.810*	0.783*

* *p* value < 0.05

** *p* value < 0.01

### Pollen production

**Fig. 3:**
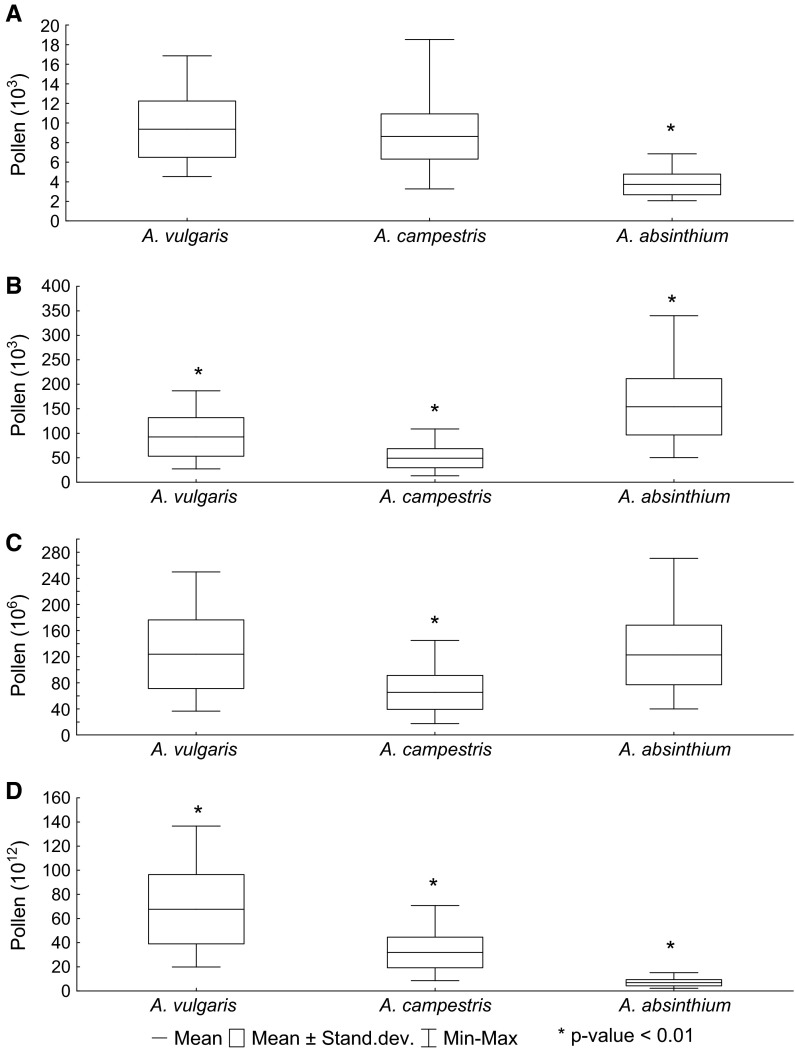
Pollen production of *Artemisia* species in Poznań estimated per flower (**a**), inflorescence (**b**), plant (**c**) and species in the study area (**d**). Statistical significance was examined by K–W ANOVA and Newman–Keuls *post* *hoc* test

The highest amounts of total pollen per single plant were produced by *A. vulgaris* and *A. absinthium* (mean = 123.8 × 10^6^ and 122.7 × 10^6^, respectively), whereas *A. campestris* produced considerably less pollen (65.3 × 10^6^) (Fig. 3c). In the case of *A. vulgaris*, such high pollen production was related to both the high number of pollen grains produced by a single flower (mean = 9.4 × 10^3^ pollen grains) and the high number of inflorescences produced by a single plant (mean = 1338 inflorescences) (Table 3). Although a single flower of *A. absinthium* produced significantly less pollen (mean = 3.7 × 10^3^, *p* < 0.001, K–W and Newman–Keuls test), the relatively high total pollen production per plant is due to the greater number of flowers in one inflorescence (mean = 42) in comparison with the other *Artemisia* species (10 for *A. vulgaris* and six for *A. campestris*) (Fig. 3; Table 3).

**Table 3:** Quantity characteristics of flowers and inflorescences

Taxon	Descriptive statistic	No. of inflorescences per plant	No. of flowers per inflorescence
*A. vulgaris*	Max	5700	16
	Min	194	6
	Mean (*n* = 90)	1338	10
	SD	1199.8	2.1
*A. absinthium*	Max	3865	67
	Min	66	20
	Mean (*n* = 45)	796	42
	SD	835.3	13.0
*A. campestris*	Max	9760	9
	Min	98	2
	Mean (*n* = 90)	1330	6
	SD	1624.2	1.6

Taking into account the estimated number of individuals of each *Artemisia* species in the study area (*A. vulgaris*, 5465; *A. absinthium*, 560; and *A. campestris*, 4885 individuals within 1 % of the study area), the calculated potential production of *Artemisia* pollen exceeded 105.8 × 10^12^. As a percentage, 63.6 % of the total pollen was produced by *A. vulgaris* (67.0 × 10^12^), 30.0 % by *A. campestris* (31.9 × 10^12^) and 6.4 % by *A. absinthium* (6.9 × 10^12^) (Fig. 3d).

### Potential pollen emission

**Fig. 4:**
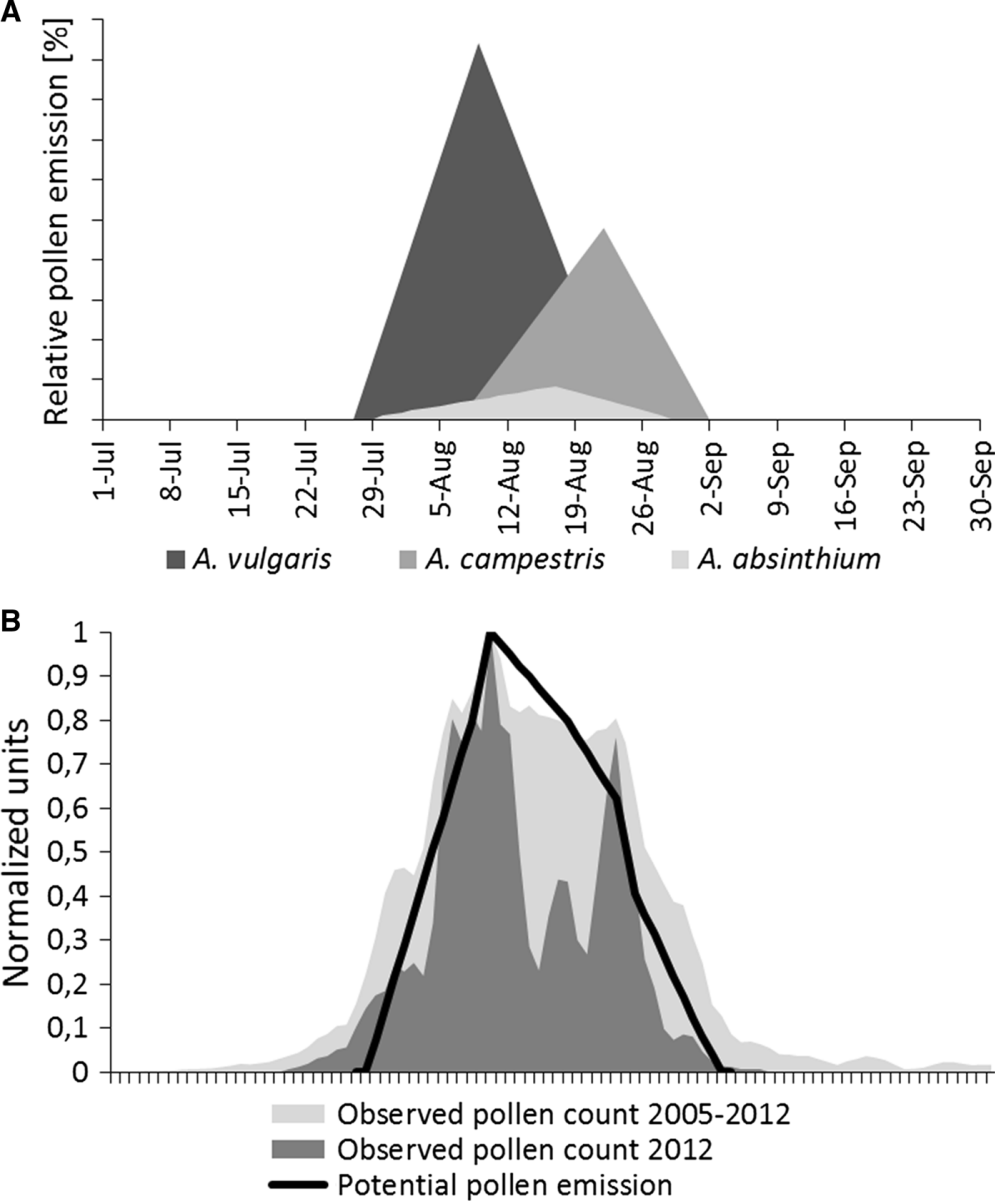
Relative pollen contribution to the pollen curve by three *Artemisia* species (**a**); normalized relationship between potential pollen emission of three *Artemisia* spp. and pollen concentration in the air (3-day moving average) (**b**)

The calculated pollen production of the three *Artemisia* species together with flowering phenology records was used to construct a theoretical curve of potential *Artemisia* pollen emission in the study area (Fig. 4). The modelled curve has a single peak in the middle of the season (flowering of *A. vulgaris*) and is slightly skewed to the right. The asymmetry of the curve is caused by the late flowering time of *A. campestris*. The potential pollen emission curve exhibited significant relationships with both the observed 8-year (2005–2012) daily average *Artemisia* pollen concentration curve and the daily average concentrations of *Artemisia* pollen recorded in the 2012 pollen season (*r*^2^ = 0.658 and *r*^2^ = 0.398, respectively, *p* < 0.05 for both). The ratio between the sum of the recorded airborne pollen counts in 2012 and the potential pollen emission in the study area was 1:51 × 10^9^. In other words, 50 billion *Artemisia* pollen grains estimated to be produced within the area of the 2 km radius corresponded to one *Artemisia* pollen grain collected at the trap level.

## Discussion

Pollen allergens produced by phylogenetically related species often exhibit a high level of cross-reactivity (Mohapatra et al. 2008). However, in some cases, the character and amount of allergenic pollen proteins may vary distinctly among related plant taxa. For instance, there is an evidence that the level of the major allergen of *Artemisia* pollen (Art v 1) may vary depending upon the *Artemisia* species (Plunkett and Jimeno 2006). Therefore, any differences observed in the pollen biology of allergenic plants may be significant for allergy sufferers. Our results, showing distinct variability in flowering phenology and pollen production among three investigated *Artemisia* species, make an important contribution to this issue.

The flowering periods of *A.vulgaris* and *A. campestris*, the two most common species in Poznań, are partially overlapped. In the first part of the season (end of July–first fortnight of August), the flowering of *A. vulgaris* was primarily observed in the study area, whereas during the second part (second fortnight of August–beginning of September), the individuals of *A. campestris* were in full flowering phase. This separation in flowering phenology was reflected in the aerobiological data. During pollen season, two peaks in *Artemisia* pollen concentrations were recorded, which coincided with the flowering times of the two mentioned *Artemisia* species. It is worth noting that the third investigated species, *A. absinthium*, was rare in the study area and therefore did not markedly influence the *Artemisia* pollen curve despite its long flowering period. The second peak observed in Poznań was not as striking as it is in south-eastern Europe, where late-flowering *Artemisia* species (possibly *A. maritima* or *A. scoparia*) may contribute over 50 % of the total annual *Artemisia* pollen (Grewling et al. 2012). However, taking into account the pollen production and abundance of *A. campestris* in the study area, we found that this species may be responsible for approximately 30 % of the total *Artemisia* pollen and therefore should be considered as an important pollen source in Poznań.

According to Gucker (2007), *A. campestris* prefers dry, sandy, open areas such as roadsides, woodland openings and old fields. In the study area, *A. campestris* was found growing in similar habitats (wastelands, sand and gravel soils), frequently creating a thick and wide conglomeration (0.5 km × 0.5 km). It has a marked ecological plasticity; however, the most appropriate conditions for this species occur in semi-arid climates (Gucker 2007). Therefore, *A. campestris* commonly occurs in the Mediterranean region, e.g. the Iberian Peninsula, where its flowering period is primarily limited to August–September (Munuera Giner et al. 1999). The later flowering of *A. campestris* in southern Europe is likely related to the hot temperatures recorded in this region, as a recent study (Grewling et al. 2012) showed that in the areas with high summer temperatures, such as the Pannonian Plain, the *Artemisia* pollen season is notably delayed. The effect of weather conditions on *Artemisia* flowering is limited not only to the timing of pollen release (Malkiewicz et al. 2014) but also to pollen production. It has been suggested (Munuera Giner et al. 1999) that scarce rainfall in the weeks before *Artemisia* pollination seriously affects plant development and, in turn, pollen production. A positive relationship between the intensity of *Artemisia* pollen seasons and the amount of rain was previously reported in Poznań (Stach et al. 2007) and Lublin in Eastern Poland (Piotrowska-Weryszko 2013). *A. campestris*, occupying primarily semi-arid habitats, may be better adapted to the shortage of rainfall than are the two other *Artemisia* species occurring in Poznań. *A. vulgaris* prefers moist, well-drained, gravel or sandy soils (Barney and DiTommaso 2003), whereas *A. absinthium* requires high moisture supply and cannot survive dry conditions (Maw et al. 1985). The physiological adaptation of *A. campestris* to semi-arid areas may be important in the context of future climate change, where further shortages of rainfall are projected for central Europe (EEA 2012). Thus, the role of *A. campestris* as a significant contributor of allergenic pollen in the region may increase.

Among the investigated *Artemisia* species, *A. vulgaris* produced the highest number of pollen grains per flower, followed by *A. campestris* and *A. absinthium*. Our calculation revealed a high variability in pollen production between individuals of the same species. For instance, the number of pollen grains per flower in *A. vulgaris* varied from approximately 4.0 × 10^3^ to over 16.0 × 10^3^ grains. Surprisingly, Piotrowska (2008) estimated in a previous study that one flower of *A. vulgaris* in Lublin (Eastern Poland) produced almost 50.0 × 10^3^ pollen grains. The observed differences may be caused by the slightly different methods used in the two studies. Piotrowska (2008) counted all pollen grains released from six anthers multiplied by the number of anthers in one flower, whereas we determined the number of pollen grains per flower according to the method proposed by Cruden (1977), i.e. by crushing a single flower in 100 µl of water and examining 10 % of the initial quantity. To obtain more robust results, our calculations were based on a much higher number of subsamples (270 = 90 flowers × 3 repetitions). It is also worth noting that Cruden’s (1977) method has been widely used in palynological studies, e.g. for interspecies comparisons of the pollen production of tree (Tormo-Molina et al. 1996), grass (Prieto-Baena et al. 2003) and weed species (Subba-Reddi and Reddi 1986). As mentioned above, our study revealed that the number of pollen grains produced per plant is highly variable and may likely depend upon many internal and external factors, such as genetic variability, weather conditions and soil content. Unfortunately, a detailed analysis of the factors affecting pollen production was beyond the scope of this study. However, as the number of produced and released pollen grains is closely related to allergy symptoms (Berger et al. 2013), further studies on this issue are highly recommended.

The flowering phenology, pollen production and plant inventory data were used to construct a theoretical curve of the potential pollen emission of *Artemisia* species within the study area. The obtained curve showed a statistically significant relationship with observed airborne pollen concentrations (*r*^2^ = 0.67, *p* < 0.05). In a similar study conducted in Cordoba, southern Spain (Hidalgo et al. 2003), where the potential pollen emission of three *Cupressus* species was investigated, the calculated coefficient of determination was slightly lower (*r*^2^ = 0.46). This difference may be caused by the fact that Hidalgo et al. (2003) sampled a larger area (5 km radius vs. 2 km in our study) and used a longer aerobiological data series (19 vs. 8 years in this study). In addition, the general characteristics of pollen grains (abilities to be transported over long distances), plant structure (height, location of inflorescences) and local biogeoclimatic factors (terrain shape, vegetation composition and changes in daily weather conditions) may also markedly affect the strength of the relationships between the airborne and ‘ground-level’ records reported in the two studies. Although the constructed model of *Artemisia* pollen emission does not perfectly reflect the variation in the aerobiological data series, it elucidates the actual contributions of certain *Artemisia* species to the airborne pollen curve. The populations of *A. vulgaris* are still the most important pollen source in the region; however, the roles of two other *Artemisia* species cannot be ignored. In particular, *A. campestris*, in the areas where it commonly occurs, should be considered an important pollen contributor that is likely responsible for allergic reactions during late summer.

## Conclusions

The timing and duration of pollination seasons as well as the pollen production of three investigated *Artemisia* species (*A. vulgaris*, *A. absinthium* and *A. campestris*) varied significantly in Poznań. Although *A. vulgaris* produced the highest number of pollen grains and was the most common within the study area, *A. campestris* also markedly influenced the total amount of *Artemisia* pollen recorded in the air, contributing approximately 30 % of the pollen total. In addition, *A. campestris* flowers in the second part of the *Artemisia* pollen season and thus is primarily responsible for the second peak of *Artemisia* pollen concentrations observed in the city. Due to its scarce occurrence in the study area, *A. absinthium* should be considered a minor pollen contributor. However, as this species produced large quantities of pollen per single individual, the airborne pollen level in the vicinity of the plants may be high and potentially risky for sensitized people. Therefore, the roles of *A. campestris* and *A. absinthium* in provoking allergy reactions should also be considered.
